# Diagnosis of Acute Gastroenteritis with Immunochromatography and Effectiveness of Rotavirus Vaccine in a Japanese Clinic

**DOI:** 10.1099/acmi.0.000085

**Published:** 2019-12-03

**Authors:** Kimiko Kawata, Toshiyuki Hikita, Sayaka Takanashi, Hiroyuki Hikita, Kaori Ogita, Shoko Okitsu, Sheikh Ariful Hoque, Tung Gia Phan, Hiroshi Ushijima

**Affiliations:** ^1^​ Division on Nursing Sciences, Midwifery, Department of Health Sciences, Faculty of Medical Sciences, Kyushu University Faculty of Medical Sciences, 3-1-1, Maidashi, Higashi-ku, Fukuoka, 812-8582, Japan; ^2^​ Hikita Pediatric Clinic, 2-7-20 Nakamchi, Kiryu city, Gunma, 376-0035, Japan; ^3^​ Department of Developmental Medical Sciences, Graduate School of Medicine, The University of Tokyo, Bunkyo, Tokyo, Japan; ^4^​ Division of Microbiology, Department of Pathology and Microbiolgy, Nihon University School of Medicine, 30-1, Oyaguchikamicho, Itabashi-ku, Tokyo, Japan; ^5^​ Cell and Tissue Culture Laboratory, Centre for Advanced Research in Sciences (CARS), University of Dhaka, Dhaka-1000, Bangladesh; ^6^​ Division of Clinical Microbiology, University of Pittsburgh Medical Center, Pittsburgh, Pennsylvania, USA

**Keywords:** Rotavirus, adenovirus, norovirus, rotavirus-vaccine, immunochromatography, vaccine effectiveness

## Abstract

Despite the well known effectiveness of two licensed live attenuated oral rotavirus (RV)-vaccines, Rotarix and RotaTeq, constant monitoring of vaccine effectiveness (VE) is essential considering the evolving power and reassortment capability of RVs. In this study, we detected RV, norovirus (NV) and adenovirus (AV) infections using immunochromatography (IC)-based kits in children with acute gastroenteritis (AGE) who attended a pediatric clinic in Kiryu city, Gunma, Japan during June, 2014–September, 2018. VEs were determined using a test-negative study design. Among 1658 AGE-children, RV, NV and AV were detected in 96 (5.8 %), 146 (8.8 %) and 46 (2.8 %) children, respectively. Interestingly, the distributions of infections were found to be associated with age and sex. Namely, RV infections were significantly higher in female (*P*=0.02) and in the 19–30 month age group children, while NV and AV infections predominated in the 13–24 month and 7–18 month age groups, respectively. The disease severity for RV and NV infections remained similar and significantly higher than that of AV infections. The VE of RV-vaccines was 49.8 % (95 % CI: 22.7 to 67.3 %) against all RV infections, which was increased up to 67.2 % (95 % CI: 35.3 to 83.4 %) against severe RV infections. RV-vaccinated children experienced less severe symptoms in RV-infections while non-RV AGE remained less serious for both RV-vaccinated and unvaccinated children. Finally, the prevalence of RV infection remained minimized (≤5.4 %) in this population since 2015. Thus, this study provided important information on distribution of major AGEs in young children and exhibited the effective role of RV vaccines in post-vaccine era.

## Introduction

Acute gastroenteritis (AGE) still remains a major global health problem mainly for infants and children [1]. There are nearly 1.7 billion childhood diarrheal cases worldwide per year that kill around 525 000 children annually, making diarrhea the second leading cause of death in children under five years of age [2]. While the vast majority of these deaths occur in developing countries, developed industrialized nations are struggling against AGE-associated significant morbidity and economic losses [3]. Viruses including rotavirus (RV) kill 215 000 children under five years of age each year [4], norovirus (NV), which kills 50 000 children below age five years of age annually, and adenovirus (AV), responsible for 1.5–5.4 % of diarrhea in children under 2 years [5], are recognized as major viral etiologies for diarrheal illness. Considering the seriousness of RV infections, two live attenuated oral RV-vaccines, Rotarix (GlaxoSmithKine) and RotaTeq (Merck) have been introduced to prevent severe RV-illness in many countries since 2006 [6]. In Japan, Rotarix and RotaTeq were introduced in November 2011 and July 2012, respectively, as a voluntary vaccination. So far, the performances of these two vaccines for providing homotypic and heterotypic protection against severe RV diarrhea appear satisfactory and they have played a significant role in reducing global deaths from 453 000 in 2008 to 215 000 in 2013 in children under five years of age [7, 8]. However, outbreaks of RV are still common in both developing and developed countries as these vaccines are more effective against development of severe RV-infection rather than protecting against milder ones [9]. Furthermore, the excellent evolving power of RVs because of the segmented genome structure and reassortment capability always poses considerable threats of waning vaccine effectiveness (VE) [9]. Continuous monitoring of VE is, therefore, crucial for understanding whether the vaccines provide enough protection against rare or emerging strains. In addition, introduction of RV vaccines may have effects on the distribution of other viral AGE that need to be investigated.

In this study, we, therefore, examined the distribution of major enteric viruses, RV, NV and AV according to age, sex, clinical features and determined the effectiveness of RV-vaccines against RV and non-RV AGE children during a period of the post-vaccine era.

## Methods

### Study sample

We collected stool samples from young children of age of 47 months or less (m) who attended Hikita Pediatric Clinic, Kiryu city, Japan with a complaint of diarrhea and/or vomiting in 3 or more episodes per day with or without nausea, fever or abdominal pain during June, 2014 to September, 2018 after obtaining written informed consents from their guardians. The demographic and clinical characteristics were recorded and used to assess the severity of the illness using a Vesikari scale, in which a score of <7, 7–10 and ≥11 out of 20 points indicated mild, moderate and severe gastroenteritis, respectively [10]. This study was approved by the ethical committees of Nihon University School of Medicine (25-13-0) and The University of Tokyo (1139).

### Immunochromatography (IC) assay

An IC-based kit, BD Rota/Adeno Examan stick (Becton, Dickinson), was used to detect RV and AV infections. NV infection was identified by ‘QuickNavi-Norovirus 2’ kit (Otsuka Pharmaceutical).

### VE determination and data analysis

The vaccination history of a child was confirmed from the ‘Maternal and Child Health Handbook’, which is provided to each mother in Japan during pregnancy. The infant was defined as ‘Vaccinated’ if he/she received the complete doses, either 2 doses of Rotarix or 3 doses of RotaTeq, at least 14 days before the onset of symptoms. A test-negative study design was used to investigate VE as described previously [11]. VE was calculated as VE=[1−adjusted odds ratio (OR)]×100. OR, adjusted for age and sex, was analyzed by logistic regression. Characteristics between two groups were compared using Pearson chi-square tests, unpaired *t*-tests, and Mann-Whitney U tests as and when required. Multiple groups were compared by one-way ANOVA with post hoc tests. All data were analyzed using SPSS-Version 16 software.

## Results

### Prevalence of Infection

Among 1658 children with AGE, 96 were positive for RV (5.8 %), 146 were positive for NV (8.8 %), and 46 were positive for AV (2.8 %) (Table 1). The mean age of AV-positive (18 months) children was significantly (*P*<0.05) lower than that of RV-positive (25 months) or NV-positive (22 months) children (Table 2). Within a range of age 0–47 m, the most vulnerable age group for RV infection was 19–30 months, whereas NV and AV infected mostly children of 13–24 months and 7–18 months, respectively. The prevalence of RV infection was significantly higher in females than in males (*P*=0.02) (Table 2).

**Table 1.** Year-wise distribution of NV, RV and AV infections in the children

Year	Total samples	RV	NV	AV
Jun, 2014–Jun, 2015	376	42 (11.2)	57 (15.2)	18 (4.8)
Jul, 2015–Jun, 2016	545	21 (3.9)	56 (10.3)	14 (2.6)
Jul, 2016–Jun, 2017	522	28 (5.4)	20 (3.8)	13 (2.5)
Jul, 2017Sept, 2018	215	5 (2.3)	13 (6.0)	1 (0.5)
Total	1658	96 (5.8)	146 (8.8)	46 (2.8)

RV, Rotavirus; NV, Norovirus; AV, Adenovirus.

**Table 2.** Characteristics of infections

Characteristics*^a^*	RV-positive AGE (*n*=96)	NV-positive AGE (*n*=146)	AV-positive AGE (*n*=46)
Age in months: mean±sd	25.3±10	22.1±10	17.8±10
*P*	RV vs NV=0.054	NV vs AV=0.041*	AV vs RV=0.0001*
Age group: *n* (%)			
0–6 m (*n*=92)	1 (1)	2 (1.4)	1 (2.2)
7–12 m (*n*=323)	11 (11.5)	26 (17.8)	16 (34.8)
13–18 m (*n*=374)	14 (14.6)	39 (26.7)	16 (34.8)
19–24 m (*n*=274)	21 (21.9)	32 (21.9)	5 (10.9)
25–30 m (*n*=220)	21 (21.9)	15 (10.3)	1 (2.2)
31–36 m (*n*=160)	14 (14.6)	10 (6.8)	3 (6.5)
37–42 m (*n*=123)	8 (8.3)	14 (9.6)	2 (4.3)
43–48 m (*n*=92)	6 (6.2)	8 (5.5)	2 (4.3)
Sex: *n* (%)			
Male (*n*=871)	39 (40.6)	77 (52.7)	27 (58.7)
Female (*n*=787)	57 (59.4)	69 (47.3)	19 (41.3)
*P*	0.02*	1	0.45
Clinical features: median (Q1Q3)			
Maximum Number Stools per day	3 (2–5)	2 (1–4)	3 (2–5)
Diarrhea Duration (days)	2 (2–4)	2 (1–4)	3 (2–4)
Maximum Number Vomiting Events per day	2 (0–5)	3 (1–5)	0 (0–1)
Vomiting Duration (days)	1 (0–2)	1 (1–2)	0 (0–1)
Temperature	38.3 (37.6–39.0)	37.1 (36.7–37.8)	37.3 (37.0–38.1)
Dehydration n (%)			
No dehydration	37 (38.5 %)	49 (33.6 %)	32 (69.6 %)
1–5 %	40 (41.7 %)	69 (47.3 %)	12 (26.1 %)
>6 %	19 (19.8 %)	28 (19.2 %)	2 (4.3 %)
Treatment: n (%)			
No	37 (38.5)	49 (33.6)	32 (69.6)
Yes (Rehydration, Hospitalization)	59 (61.5)	97 (66.4)	14 (30.4)
Vesikari Score: mean±sd	8.9±4.1	8.4±3.8	6.0±3.1
*P*	RV Vs NV=0.78	NV Vs AV=0.001*	AV Vs RV=0.000*
Severity: n (%)			
Mild	31 (32.3)	48 (32.9)	29 (63.0)
Moderate	22 (22.9)	56 (38.4)	13 (28.3)
Severe	43 (44.8)	42 (28.8)	4 (8.7)

*a*, Multiple comparisons of Means (±sd) were performed by one-way ANOVA and post hoc analysis, groups were compared by Pearson Chi-square test.

*Indicate statistically significant values.

### Clinical features of Infection

The frequency of diarrhea remained almost similar in all these three infections, whereas the duration and frequency of vomiting were found to be lower in AV-positive children (Table 2). The temperature of RV-infected children remained higher (median 38.3 °C) compared with that in NV- and AV-positive children. The majority of children with RV or NV infection, but not those with AV infection suffered dehydration problems and needed rehydration treatment. The Vesikari severity score of RV- or NV- infected children remained significantly higher than that of AV-infected children. A large number of children with RV infection had severe infection whereas the majority of NV and AV-infected children had moderate and mild AGE infections, respectively (Table 2).

### Vaccine Effectiveness (VE)

Out of 1658 children with AGE, 898 (54 %) children completed full doses of RV-vaccination, 5 (0.3 %) had received incomplete doses, and 732 children did not receive any doses. Vaccine history remained unknown in 23 children. Here, children with unknown or incomplete RV vaccination history were excluded from the analysis of VE.

Among 95 RV-positive children, 36 (38 %) were vaccinated, resulting in a VE of 49.8 % (95 % CI: 22.7 to 67.3 %; *P*=0.002) (Table 3). Excluding the number of mildly infected children, among 64 RV-positive children with moderate/severe infections (severity score ≥7), 19 (30 %) were vaccinated: VE was calculated as 64.5 % (95 % CI: 38.6 to 79.5 %; *P*<0.001). Similarly, VE was evaluated as 67.2 % (95 % CI: 35.3 to 83.4 %; *P*=0.001) against severe infections (severity score ≥11). Our data, thus, indicate that these vaccines remained significantly effective against RV infection and VE was increased with disease severity. As expected and shown in Table 3, RV vaccine had no significant effect on preventing NV (*P*>0.05) and AV (*P*>0.05) infections.

**Table 3.** Effectiveness of complete doses of RV vaccines against different severity of RV infection

	Cases Number	Vaccinated (percentage) among cases	Control Number	Vaccinated (percentage) among controls	Adjusted VE (95 % CI)	*P*
RV-infection						
Severity ≥1	95	36 (38 %)	1535	862 (56 %)	49.8 % (22.7 % to 67.3%)	0.002*
Severity ≥7	64	19 (30 %)	1535	862 (56 %)	64.5 % (38.6 % to 79.5%)	0.000*
Severity ≥11	43	12 (28 %)	1535	862 (56 %)	67.2 % (35.3 % to 83.4%)	0.001*
NV-infection						
Severity ≥1	142	73 (51 %)	1488	825 (55 %)	14.3 % (-21 % to 39.4%)	0.382
Severity ≥7	96	49 (51 %)	1488	825 (55 %)	16.5 % (-26.4 % to 45%)	0.394
Severity ≥11	41	20 (49 %)	1488	825 (55 %)	28.1 % (-34 % to 61.5%)	0.3
AV-infection						
Severity ≥1	46	26 (56.5 %)	1584	872 (55 %)	0 % (-81 % to 45%)	0.999
Severity ≥7	17	8 (47 %)	1584	872 (55 %)	35.6 % (-68.6 % to 75.4%)	0.370
Severity ≥11	4	0 (0 %)	1584	872 (55 %)	–	–

VE (vaccine effectiveness) was determined against RV, NV and AV infections (Severity Score ≥1,≥7 and≥11) by (1−adjusted OR)×100. OR adjusted for age with 95 % CI (confidence interval) and probability (*P*)-value was determined by binary logistic regression adjusted for age and sex.

*Indicate statistically significant values.

### Effect of RV vaccination on clinical outcomes

In 95 RV-positive children, the disease severity score was significantly lower (*P*=0.04) in RV-vaccinated children than that in unvaccinated children. Vaccinated children experienced significantly lower numbers of vomiting events per day, fewer dehydration problems and less necessity for rehydration compared with unvaccinated children (Table 4).

**Table 4.** Comparison of clinical characteristics in vaccinated and unvaccinated RV-positive AGE cases

Characteristics^*a*^	RV-positive AGE (*n*=95)			RV-negative AGE (*n*=1535)		
	Vaccinated	Unvaccinated	*P*	Vaccinated	Unvaccinated	*P*
Clinical features						
Maximum Number Stools per day	2 (1–5)	3 (2–5)	0.054	3 (1–5)	3 (1–5)	0.937
Diarrhea Duration (days)	2 (0.2–4)	3 (2–4)	0.069	2 (1–4)	2 (1–4)	0.119
Maximum Number Vomiting Events per day	1 (0–3)	3 (1–6)	0.033*	0 (0–1)	0 (0–2)	0.011*
Vomiting Duration (days)	1 (0–2)	1 (1–2)	0.407	0 (0–1)	0 (0–1)	0.039*
Temperature	38.4±0.8	38.2±1.0	0.375	37.3 (36.9–38.5)	37.5 (36.9–38.7)	0.031*
Dehydration						
No	20 (56 %)	17 (29 %)	0.01*	577 (67 %)	440 (65 %)	0.550
Yes (1–5 %,>6 %)	16 (44 %)	42 (71 %)		285 (33 %)	233 (35 %)	
Treatment						
No	20 (56 %)	17 (29 %)	0.01*	577 (67 %)	440 (65 %)	0.550
Yes (Rehydration)	16 (44 %)	42 (71 %)		285 (33 %)	233 (35 %)	
Vesikari Score	7.9±4.2	9.7±4.0	0.04*	5.9±3.5	6.2±3.7	0.063

*a*, Means±sd were compared by unpaired t-tests, variables in non-parametric distributions (medians (Q1–Q3) were compared by Mann-Whitney U tests, groups were compared by Pearson Chi-square test.

*Indicate statistically significant values.

Finally, we examined whether RV-vaccination has any advantage in RV-negative AGE children. In fact, clinical symptoms of RV-negative children were less severe than those of RV-positive children. Although some statistically significant differences appeared between vaccinated and unvaccinated children in the RV-negative AGE group, such statistical differences had no clinical importance since the average values remained similar between vaccinated and unvaccinated groups (Table 4). No significant differences were found for dehydration problems, treatment requirement and disease severity in vaccinated and unvaccinated children.

## Discussion

This study examines the distribution of RV, NV and AV infections according to age, sex and clinical manifestations in Japanese children in Kiryu city during 2014–2018, nearly 2–3 years after the introduction of RV-vaccine in Japan as a voluntary vaccination. One main objective of this study was to examine the role of RV-vaccines against viral AGE in outpatient pediatric clinics where patients attend usually. Notably, RV-vaccines, both Rotarix and RotaTeq, were originally designed to provide protection against severe diseases caused by common circulating strains and are repeatedly reported to be very much effective, mainly against hospitalization as well as moderate to severe RV infections [9]. Previously, we have demonstrated that RV-vaccines do not provide enough protection against mild RV infections resulting in outbreaks, but they play a crucial role in limiting disease severity [9, 11].

In the present study, complete RV vaccine coverage was found in 54 % of children in Gunma prefecture, which remained consistent with our earlier report of that in Shizuoka prefecture [9]. NV infections remained the most common (8.8%), followed by RV (5.8%) and AV infections (2.8%). However, these prevalence rates are often found to be higher when evaluated by reverse transcription polymerase chain reaction (RT-PCR). In fact, IC is not as sensitive as RT-PCR. Nevertheless, the trend of infections detected here, i.e., the most common infection is NV followed by RV and AV, remained compatible with many other earlier reports [12]. Indeed, NV infection has become more frequent in Japan since 2003 before the introduction of RV-vaccine [12, 13]. A review on epidemiology of gastroenteritis viruses in Japan till 2015 reported that the prevalence of NV was between 13.2 and 40.7 %, while that of RV and AV were 4.0–42.2 and 0.6–12.5 %, respectively [13, 14]. Previously, we have demonstrated that the prevalence of RV in Japan has been declining to 4.0 % in 2013–2014 from 17.9 % in 2011–2012 and 22.1 % in 2012–2013 after the introduction of RV-vaccine [14]. In agreement with previous reports, if we look at the year-wise distribution of RV infections in the present study, RV infection was 11.2 % in 2014–2015, followed by 3.9 % in 2015–2016, 5.4 % in 2016–2017 and 2.6 % in 2017–2018 in this pediatric clinic (Table 1). Our data, thus, reveals that RV-vaccine remained very much effective to keep RV infection minimized in this population.

Importantly, in the present study, we found association of RV, NV or AV infections with age and sex (Table 2) which has hardly been reported before. One possible explanation is that such studies are usually carried out on young children under 5 years (60 m) of age while in the present study we considered only children between 0 and 47 months since the time difference between RV-vaccine introduction in Japan and the beginning of the study was 47 months only. Our data indicated that within the 0–47 m age range, 19–30 m (mean 25 m), 13–24 m (mean 22 m) and 7–18 m (mean 18 m) age groups were the most vulnerable for RV, NV and AV infections, respectively (Table 2). Another important plausible explanation is that RV-vaccine may remain very much effective at the early stage of vaccination after the administration within the first 6 months of life. Therefore, children with older age around 25 months became more vulnerable to RV infection while younger children from 7 to 24 months became more susceptible to other viral AGE.

Here, RV infections were found to be significantly higher in females (Table 2) which is also very interesting. Careful observation reveals that the significant differences in acquiring RV infection in females mainly occurred in the RV-vaccine negative population (*P*=0.014). In fact, RV-vaccine negative female children had two times more chance of being infected with RV than unvaccinated males (OR: 1.993; 95 % CI: 1.15–3.45). The severity scores were almost similar for both RV and NV infections which were significantly higher than that of AV infections (Table 2).

When we examined VE against RV-infections, RV-vaccine was found to be significantly (*P*<0.002) effective against all (severity ≥1), moderate/severe (severity ≥7), and severe (severity ≥11) RV infections (Table 3). As expected, no significant effectiveness of RV vaccine was determined against NV and AV infections (Table 3). RV-vaccinated children experienced less severe symptoms than unvaccinated children during RV infection (Table 4) which remained consistent with our previous findings [9]. However, both RV-vaccinated and unvaccinated children experienced similar and mild clinical conditions in RV-negative AGE (Table 4). The statistically significant differences detected for some clinical features between vaccinated and unvaccinated RV-negative AGE children as shown in Table 4 were mainly because of small differences in the large number of samples which have no clinical importance.

One important limitation of this study is that the infection was detected by IC only, and the result was not confirmed by molecular analysis such as PCR/RT-PCR. Although detection with IC is not as sensitive or specific as molecular detection, a comparison of VEs determined by IC and RT-PCR indicated that VEs evaluated by IC remain comparable with those detected by PCR/RT-PCR, particularly when detecting moderate and severe infections (data not shown). Another limitation of the present study is that the study has included patients from a single clinic representing only a small area of Kiryu city of Gunma prefecture. However, the large sample size as well as the longer study time has increased the strength of this study. No patient in our study required hospitalization, indicating that the patients were not clinically so seriously affected. Thus, the VEs determined here against RV infections remained unique to these patients.

In conclusion, this study confirms significant effectiveness of RV vaccine against mild, moderate and/or severe RV infections. It also has confirmed the remarkable role of RV vaccine in alleviating the symptoms in RV-infected patients. The effect of RV vaccine against NV and AV infections and/or symptom alleviation was examined and found not to be clinically important. Regular monitoring of VE is necessary to keep the RV vaccination program successful and effective against emerging and newer RV genotypes.

## References

[1] Hoque SA, Thongprachum A, Takanashi S, Mostafa SM, Saito H (2019). Alarming situation of spreading enteric viruses through sewage water in Dhaka City: molecular epidemiological evidences. Food Environ Virol.

[2] WHO (2017). Diarrhoeal disease. https://www.who.int/news-room/fact-sheets/detail/diarrhoeal-disease.

[3] Stuempfig ND, Seroy J (2019). Viral Gastroenteritis. StatPearls [Internet].

[4] Pecenka C, Parashar U, Tate JE, Khan JAM, Groman D (2017). Impact and cost-effectiveness of rotavirus vaccination in Bangladesh. Vaccine.

[5] Oude Munnink BB, van der Hoek L (2016). Viruses causing gastroenteritis: the known, the new and those beyond. Viruses.

[6] Abou-Nader AJ, Sauer MA, Steele AD, Tate JE, Atherly D (2018). Global rotavirus vaccine introductions and coverage: 2006 - 2016. Hum Vaccin Immunother.

[7] Tate JE, Burton AH, Boschi-Pinto C, Steele AD, Duque J (2012). 2008 estimate of worldwide rotavirus-associated mortality in children younger than 5 years before the introduction of universal rotavirus vaccination programmes: a systematic review and meta-analysis. Lancet Infect Dis.

[8] Moyo SJ, Blomberg B, Hanevik K, Kommedal O, Vainio K (2014). Genetic diversity of circulating rotavirus strains in Tanzania prior to the introduction of vaccination. PLoS One.

[9] Hoque SA, Kobayashi M, Takanashi S, Anwar KS, Watanabe T (2018). Role of rotavirus vaccination on an emerging G8P[8] rotavirus strain causing an outbreak in central Japan. Vaccine.

[10] Lewis K (2011). Vesikari clinical severity scoring system manual: PATH, A catalyst for global health.

[11] Hoque SA, Islam MT, Kobayashi M, Takanashi S, Anwar KS (2018). Our response to the letter to the editor. Vaccine.

[12] Thongprachum A, Takanashi S, Kalesaran AF, Okitsu S, Mizuguchi M (2015). Four-year study of viruses that cause diarrhea in Japanese pediatric outpatients. J Med Virol.

[13] Thongprachum A, Khamrin P, Maneekarn N, Hayakawa S, Ushijima H (2016). Epidemiology of gastroenteritis viruses in Japan: prevalence, seasonality, and outbreak. J Med Virol.

[14] Takanashi S, Thongprachum A, Okitsu S, Nishimura S, Kobayashi M (2017). Molecular epidemiological traits of group a rotaviruses in Japanese children during transitional period of rotavirus vaccine implementation, 2011 - 2014. Clin Lab.

